# CD117 (c-Kit) Is Expressed During CD8^+^ T Cell Priming and Stratifies Sensitivity to Apoptosis According to Strength of TCR Engagement

**DOI:** 10.3389/fimmu.2019.00468

**Published:** 2019-03-15

**Authors:** Guido Frumento, Jianmin Zuo, Kriti Verma, Wayne Croft, Pradeep Ramagiri, Frederick E. Chen, Paul Moss

**Affiliations:** ^1^Institute of Immunology and Immunotherapy, University of Birmingham Birmingham, United Kingdom; ^2^NHS Blood and Transplant, Birmingham, United Kingdom; ^3^Centre for Computational Biology, University of Birmingham Birmingham, United Kingdom; ^4^Centre for Clinical Haematology, University Hospitals Birmingham NHS Foundation Trust Birmingham, United Kingdom; ^5^Royal London Hospital, Barts Health NHS Trust London, United Kingdom

**Keywords:** CD117, c-kit, stem cell factor, T cells, apoptosis, galectin-1

## Abstract

CD117 (cKit) is the receptor for stem cell factor (SCF) and plays an important role in early haemopoiesis. We show that CD117 is also expressed following priming of mature human CD8^+^ T cells *in vitro* and is detectable following primary infection *in vivo*. CD117 expression is mediated through an intrinsic pathway and is suppressed by IL-12. Importantly, the extent of CD117 expression is inversely related to the strength of the activating stimulus and subsequent engagement with cell-bound SCF markedly increases susceptibility to apoptosis. CD117 is therefore likely to shape the pattern of CD8^+^ T cell immunodominance during a primary immune response by rendering cells with low avidity for antigen more prone to apoptosis. Furthermore, CD117^+^ T cells are highly sensitive to apoptosis mediated by galectin-1, a molecule commonly expressed within the tumor microenvironment, and CD117 expression may therefore represent a novel and potentially targetable mechanism of tumor immune evasion.

## Introduction

Stem cell factor (SCF) is a cytokine growth factor expressed by a range of stromal cells and binds to its cognate receptor CD117, a tyrosine kinase also called cKit. CD117 is expressed on germ cells, hematopoietic stem cells, and early hematopoietic progenitors. Expression is lost during cellular differentiation although CD117 is retained on mature mast cells and melanocytes (1, 2). Cellular engagement of CD117 by SCF potentiates a number of cytokine-dependent signaling pathways, promoting a range of functions in germ and stem cells, including survival, proliferation, differentiation, and migration (1, 2).

SCF is produced in both soluble and membrane-bound forms which elicit differential quantitative and qualitative responses in target cell signaling following binding to CD117 (2). Furthermore, two transmembrane isoforms, SCF^248^ and SCF^220^, are generated by alternative splicing of exon 6, which encodes the proteolytic cleavage site that releases the soluble isoform (1, 2). Membrane-bound SCF induces a much stronger stimulatory effect than soluble SCF (1) as binding of the latter isoform triggers rapid internalization and degradation of CD117, ultimately resulting in reduced signaling (3, 4).

Mice lacking either SCF or CD117 are not viable and die either *in utero* or in the early postnatal period (1). Hypomorphic mutations resulting in reduced expression or function of the two proteins have less dramatic effects including macrocytic anemia, depigmentation, reduced fertility, or decreased number of mast cells (1). Similar traits are also observed in mice engineered to express only the soluble form of SCF (5). SCF-CD117 interactions play an important role in early lymphopoiesis where SCF supports the survival and expansion of CD3^−^/CD4^−^/CD8^−^ thymocytes (6) but there is little evidence for a role in regulation of the mature T cell subset. Indeed, CD117 or SCF deficient mice show grossly normal T cell development although the αβ/γδ T cell ratio within intraepithelial lymphocytes of the intestinal epithelium is increased due to higher numbers of CD4^+^/CD8^+^ αβT cells (7). Soluble SCF was reported to potentiate the allogeneic mixed lymphocyte reaction (8), but there is no direct evidence, either in mice or in humans, of mature T cells expressing CD117.

We observed CD117 mRNA expression within recently activated human naïve CD8^+^ T cells and this unexpected finding prompted us to investigate the role of CD117 expression in human mature T lymphocytes. Our results demonstrate that CD117 expression is induced on naive T cells following initial activation. Moreover, the magnitude of this expression is inversely related to the strength of the activating stimuli and CD117 expression is associated with both reduced proliferation and differentiation and an increased sensitivity to pro-apoptotic stimuli. These findings reveal a role for CD117 in shaping CD8^+^ T cell immunodominance and, as tumors frequently evolve mechanisms to potentiate T cell apoptosis, as a potential novel mechanism of immune evasion in cancer.

## Materials and Methods

### T Cell Separation and Culture

PBMC and CBMC were obtained by Ficoll separation. Enriched naïve CD8^+^ T cells were isolated with the Naïve CD8^+^ T Cell Isolation Kit (Miltenyi Biotech, Bergisch Gladbach, Germany). CD8^+^ T_CM_ and T_EM_ cells were negatively isolated from CD8^+^ T cells enriched with the CD8^+^ T Cell Isolation Kit (Miltenyi) by removal of CD45RA^+^ cells with anti-CD45RA-APC and anti-APC MicroBeads (Miltenyi). CD117^+^ and CD117^−^ cells were obtained from enriched CD8^+^ T cells using anti-CD117-APC and anti-APC MicroBeads (Miltenyi). MJS cells were removed using anti NGFR/APC (clone ME20.4, BioLegend, San Diego, CA, USA) and anti-APC MicroBeads. The purity of the enriched samples was checked by flow cytometry. Cells were cultured in RPMI 1640 supplemented with 10% FCS.

### SCF Gene Transfection

Retroviral constructs were engineered by cloning SCF^220^ into the pLZRS retroviral vector. Immediately downstream from the inserted gene was an IRES and the truncated nerve growth factor (ΔNGFR) gene. Vesicular stomatitis virus-pseudotyped retrovirus particles were produced in GP2-293 cells co-transfected with the pVSV-G envelope vector. Virus in the culture supernatant at 72 h was used to infect overnight 5 × 10^5^ MJS cells. The outcome of transduction was checked by flow cytometry (Figure S1A).

### T Cell Activation and Treatment

T cells were activated with either of the following stimuli. Anti-CD3 (CD3): cells were incubated with 66 ng/mL anti-CD3 antibody (OKT3), plus 300 U/mL IL-2 (Miltenyi); cells were activated in this way throughout the study, unless otherwise indicated. CD3/CD28 beads: Dynabeads T Activator CD3/CD28 beads (Life Technologies, Grand Island, NY, USA) were incubated with cells at 1:1 ratio in the presence of 30 U/mL IL-2. Phytohemagglutinin (PHA): cells were incubated with 1% PHA M (Life Technologies), plus 50 U/mL IL-2. Phorbol 12-myristate 13-acetate plus ionomycin (PMA-ionomycin): Cell Stimulation Cocktail (eBioscience, San Diego, CA, USA) was added at 1:500 ratio, plus 30 U/mL IL-2.

After activation, half of the culture medium was replaced thrice a week with new medium plus 50 U/mL IL-2, unless otherwise indicated.

In some experiments cells were activated with anti CD3 plus IL-2, at day 5 washed, and from then on maintained in IL-2, IL-6, IL-7, IL-12, IL-15, or IL-21 (all from Miltenyi) resupplying the cells trice a week.

Dexamethasone (Enzo Life Sciences, Farmingdale, NY, USA) and galectin-1 (R&D Systems, Minneapolis, MN, USA) were used to induce apoptosis in T cells. CD117^+^ cells were re-stimulated with anti CD3 plus IL-2 as indicated above, and after 3 days dexamethasone or galectin-1 was added. Apoptosis was measured after 24 h. The pan-caspase inhibitor Z-VAD-FMK (R&D Systems) was added 1 h prior to dexamethasone to inhibit caspase activity.

Soluble SCF (R&D Systems) was added to CD117^+^ cells at the time of activation with anti CD3 plus IL-2, and apoptosis and proliferation were measured at day 1 and day 3, respectively.

3 × 10^4^ MJS cells, either SCF-transduced or mock-transduced, were co-incubated at 1:10 ratio with CD117^+^ cells at day 3 after re-activation in flat bottom 96 well plates in the presence of galectin-1. Apoptosis was measured after 24 h. In some experiments, CD117^+^ cells were pre-incubated overnight with soluble SCF 200 ng/mL before co-culture with MJS cells and maintained in SCF throughout the experiment.

### Flow Cytometry Analysis

For CD117 staining, the “research-use-only” clones A3C6E2, AC126 (both from Miltenyi) and YB5.B8 (BD, San Jose, CA, USA), and the “for-*in vitro*-diagnostic-use” clone 104D2 (BD) were used.

SCF expression was detected via indirect staining using the rabbit anti SCF clone EP665Y (Abcam, Cambridge, UK) and a PE-conjugated goat anti rabbit IgG (R&D, Minneapolis, MN, USA). Staining with PE-conjugated tetramers identified CD8^+^ T cells specific for the EBV-derived, HLA-A2-restricted peptides YVL, GLC, and CLGGLLTMV (CLG), and B8-restricted peptides FLRGRAYGL (FLR) and RAKFKQLL (RAK).

For intracellular cytokine staining, CD117^+^ and CD117^−^ CD8^+^ T cells were reactivated with PHA+ionomycin and 1 h later 1 μL/mL Golgi Stop (BD) was added. After overnight incubation cells were fixed and permeabilized using the FIX&PERM kit (ADG, Kaumberg, Austria), then stained with the following antibodies: IL-2/Fitc clone 5344.11, TNF/PE-Cy7 clone MAb11, and IFNγ/APC clone B27 (all from BD).

For intracellular Nur77 staining the anti Nur77/PE clone 12.14 (Thermofisher Scientific, Waltham, MA, USA) was used (9).

Details on the other antibodies used are provided in Table S1.

Gating strategy involved selection of single cells and use of a “dump channel” including 7-aminoactinomycin D (BD) and PerCP-conjugated anti CD14, CD16, and CD19. Samples were read on a FACSCanto II (BD).

### Measurement of Cell Proliferation and Apoptosis

The enumeration of cells in the different phases of cell cycle or in apoptosis was performed using propidium iodide (PI), as previously described (10). Briefly, the cell pellet was incubated for 30 s in PBS plus 0.1% Triton × 100. Afterward, PBS plus PI 50 μg/mL and RNase 500 μg/mL was added, and samples were analyzed after 30 min at room temperature. Examples of the histograms obtained are provided in Figure S1B.

In some instance data on proliferation were confirmed by staining cells for 2 min with 10^−6^ M carboxyfluorescein succinimidyl ester (CFSE) prior to activation, while data on apoptosis were confirmed by measuring the uptake of Annexin V with the TACS Apoptosis Detection Kit (Trevigen, Gaithersburg, MD, USA) following manufacturer's instructions.

### Microarray Analysis

Gene expression analysis was performed on cells from 3 UCB samples, i.e., 3 CD8^+^ T_N_ samples and three samples of CD8^+^ T_N_ cells which had been activated with the CD3 protocol, and after 6 days maintained for further 8 days in 25 ng/mL IL-7. RNA extraction was performed using RNeasy columns (Qiagen, Hilden, Germany). Source RNA was confirmed as high quality by use of a Bioanalyzer 2,100 (Agilent Technologies, Santa Clara, CA, USA). RNA Integrity Numbers of 6.0 were confirmed for all samples using a RNA 6,000 Pico Chip kit (Agilent). Twenty-five nanogram of each source sample RNA was labeled with Cy3 dye using the Low Input Quick Amp Labeling Kit (Agilent). A specific activity of >6.0 was confirmed by measurement with a spectrophotometer. Six hundred nanogram of labeled RNA was hybridized to SurePrint G3 Human 8 × 60K microarray slides (Agilent). After hybridization, slides were scanned with a High Resolution C Scanner (Agilent), using a scan resolution of 3 mm. Feature extraction was performed using Feature Extraction Software (Agilent), with no background subtraction. Extracted data were normalized using the R 3.0.1 software environment with the limma 3.16.8 analysis package (11). Log transformed expression values were analyzed using two class paired analysis, SAM v4.01.

### Signal Pathway Analysis

Total RNA was extracted using RNAeasy columns following the manufacturer's protocol (Qiagen, Hilden, Germany). The quality and quantity of RNA was measured on Nanodrop-1,000 (Thermofisher). 0.5 μg of total RNA was used for cDNA synthesis using RT2 First strand kit (Qiagen). RT^2^ SYBR Green qPCR mastermix with RT^2^ Profiler PCR Array (PAHS-014ZA, Qiagen) were used in the gene expression studies. Data analysis of the PCR array was performed with Qiagen's web based software and further confirmed with R programme using ΔΔCt values. Any gene with a Ct value >35 was considered undetectable. The raw data were normalized to the average of five housekeeping genes, namely ActB, B2M, GAPDH, HPRT1, and RPL90.

### Statistics

Differential gene expression was accepted as statistically significant if the False Discovery Rate was below 5% and the fold change in gene expression was above 2. Functional and network analysis was performed on the pool of significant genes using Ingenuity Pathway Analysis (Qiagen).

To identify the pathways enriched in the significantly differentially expressed genes in the signal pathway analysis we performed gene set enrichment analysis in R programme using the Hallmark pathways data set obtained from the Molecular Signatures Database (12). *P*-value and FDR q-value for the above analyses were obtained using R programme to determine statistical significance of enrichment.

The other data were analyzed using Student's *t*-test. Data are reported as mean ± 1SD throughout the text.

## Results

### CD117 Is Expressed on CD8^+^ T Cells Following Primary Activation *in vitro*

**Figure 1:**
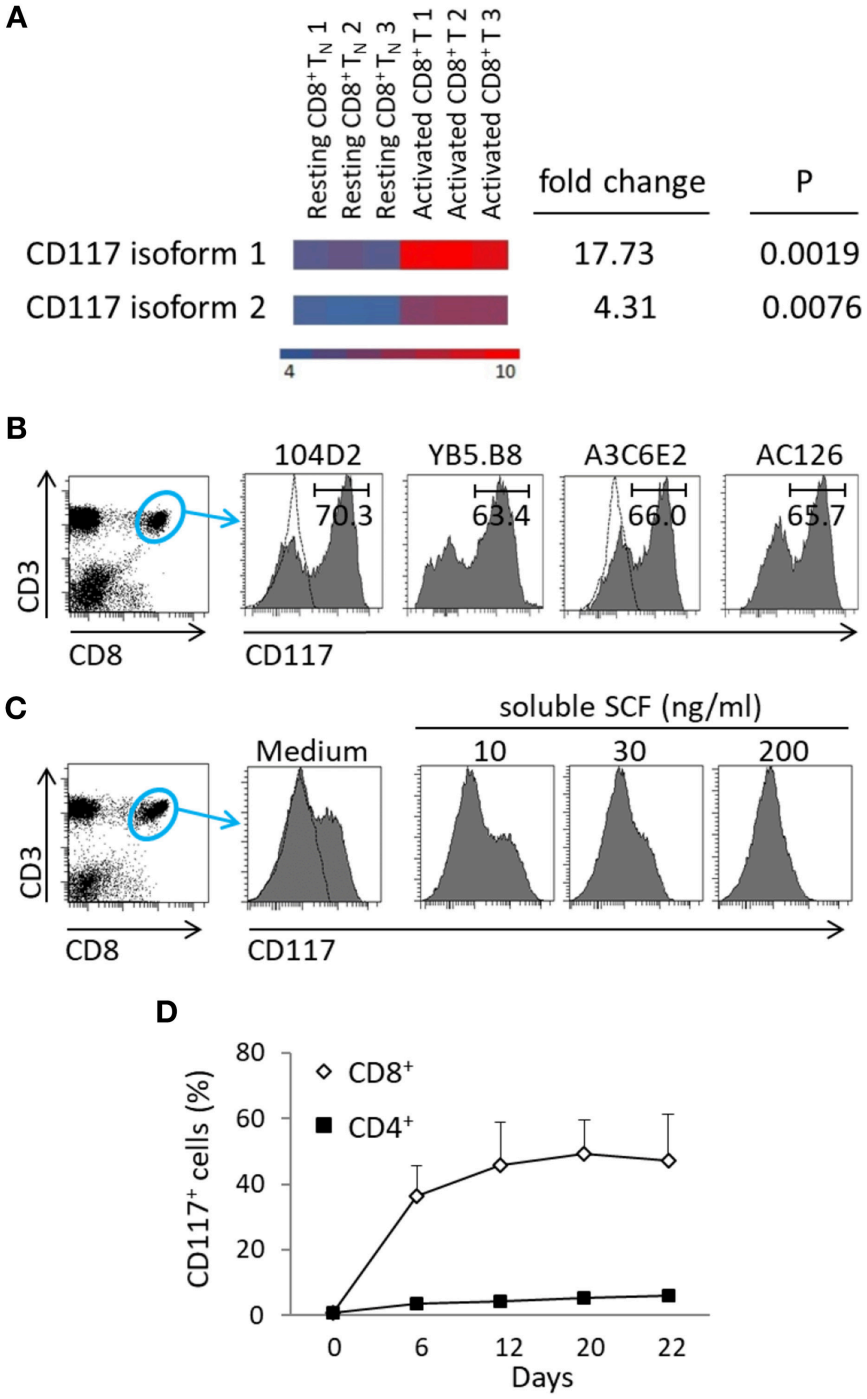
CD117^+^ expression is induced on CD8^+^ T cells following recent activation. **(A)** mRNA transcripts of both isoforms of CD117 are upregulated in enriched CD8^+^ T_N_ cells from UCB activated with anti-CD3 plus IL-2 for 5 days and then maintained in IL-7 for 2 weeks, as compared to resting T_N_ from UCB. Transcriptome analysis was performed on three different samples for each type of cells. The heat map shows down-regulated genes in blue and up-regulated genes in orange, scale: log2 normalized intensity. **(B)** CBMC were activated at day 0 and day 12 with anti-CD3 plus IL-2 and at day 21 stained with different clones of CD117-specific MoAbs, as indicated. Histograms show the results of gating on CD3^+^CD8^+^ cells. The dotted lines indicate the isotype controls, one for each company, since the antibodies are all IgG1. A single representative experiment out of three is shown. **(C)** CBMC at day 7 after activation were incubated overnight with escalating doses of soluble SCF and the expression of CD117 was then determined. Histograms show the results of gating on CD3^+^/CD8^+^ cells. A single representative experiment out of three is shown. **(D)** CBMC were activated with anti-CD3 plus IL-2 and the kinetics of CD117 expression was recorded, gated on either CD3^+^CD8^+^ or CD3^+^CD4^+^ cells. Data are from six independent cord blood samples.

Cord blood mononuclear cells (CBMC) were isolated from human umbilical cord blood (UCB) and activated through incubation with an anti-CD3 antibody. The transcriptional profile of resting and activated cells was compared and revealed differential expression of the two CD117 isoforms (13), which were highly expressed in activated cells (Figure 1A). Protein expression of CD117 was analyzed using four different clones of CD117-specific antibodies. Whilst CD117^+^ cells were not found within fresh UCB (Figure S2A), CD117^+^ subpopulations were observed with all four monoclonal antibodies within recently activated CD8^+^ T cells (Figure 1B).

Binding of CD117 with the soluble form of SCF induces a rapid internalization of the complex (3, 4) and we therefore went on to use soluble SCF to further confirm the specificity of staining. As anticipated, the incubation of activated CBMC with escalating doses of soluble SCF was shown to result in progressive reduction in the subsequent ability to detect CD117 expression on CD8^+^ T cells (Figure 1C). Interestingly, CD117 expression was detected only on recently activated CD8^+^ T cells with no expression being seen on CD4^+^ T cells (Figure 1D).

### Activation-Induced Expression of CD117 Is Seen on Naïve CD8^+^ T Cells From Both Cord Blood and Adults

**Figure 2:**
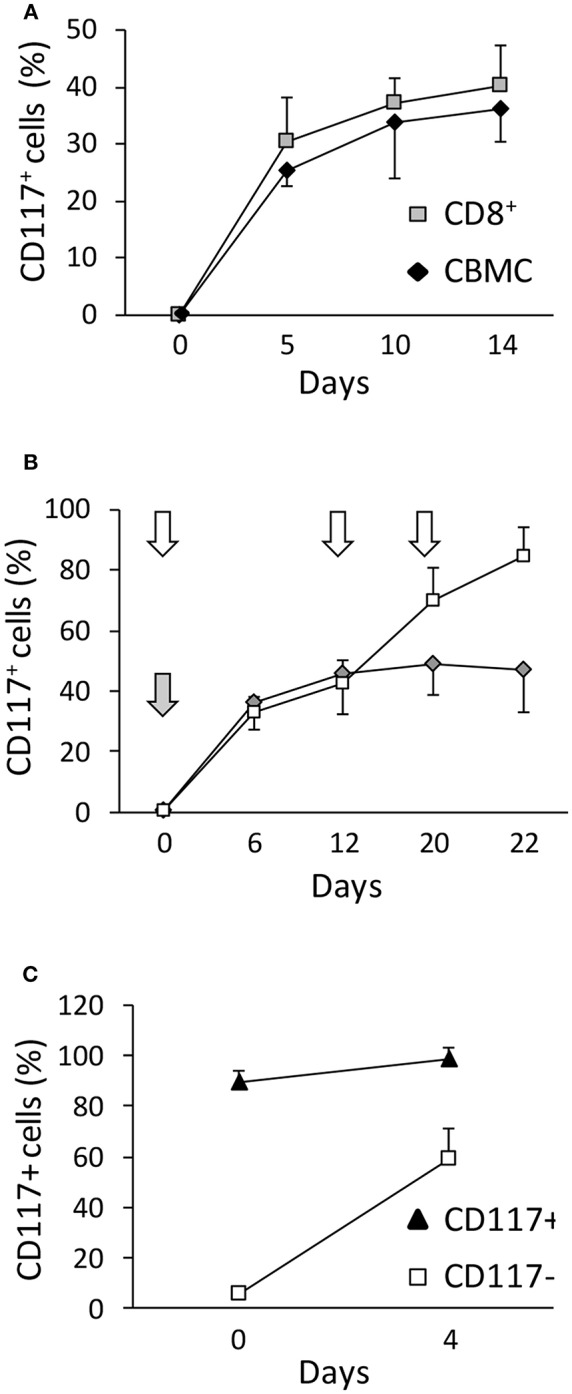
CD117^+^ is selectively expressed on CD8^+^ T cells and incremental expression is observed after serial episodes of activation. **(A)** The kinetics of activation-induced CD117 expression was determined on CBMC or CD8^+^ T cells purified from UCB by immunomagnetic separation. Gating was set on CD3^+^/CD8^+^ cells. Data are from three independent UCB units. **(B)** The level of CD117 expression was determined on CD8^+^ following either a single episode of stimulation at day 0 (gray arrow and diamonds) or after re-stimulation at day 12 and 20 (white arrows and squares). Data are from three independent CBMC samples. **(C)** CD117^+^ and CD117^−^ cells were enriched from purified CD8^+^ T cells from UCB 12 days after activation and re-stimulated with anti-CD3 plus IL-2. The level of CD117 expression is shown before and 4 days after re-activation. Data are from three independent samples.

In order to determine factors that regulate the expression of CD117 on activated CD8^+^ T cells we first investigated the potential importance of intrinsic and extrinsic cellular factors. As such, expression was evaluated on activated populations of either mononuclear cells or purified CD8^+^ T cells from UCB. Of note, expression was equivalent on both populations indicating that the ability to express CD117 upon activation is intrinsic to CD8^+^ T cells and does not require signaling from other mononuclear leukocytes (Figure 2A).

We next went on to examine the influence of serial episodes of activation on the profile of CD117 expression. Interestingly, it was clear that not all CD8^+^ cells expressed CD117 after one round of activation (Figure 2B) but this percentage increased after repeated re-stimulation. This phenomenon was not due to preferential proliferation of the CD117^+^ subset or spontaneous death of CD117^−^ cells, but instead related to differences among the cells in the response kinetics to activation, such that some cells required repeated rounds of activation before expressing CD117. Indeed, half of the cells that had not expressed CD117 after the first activation became CD117^+^ after re-stimulation (Figure 2C).

**Figure 3:**
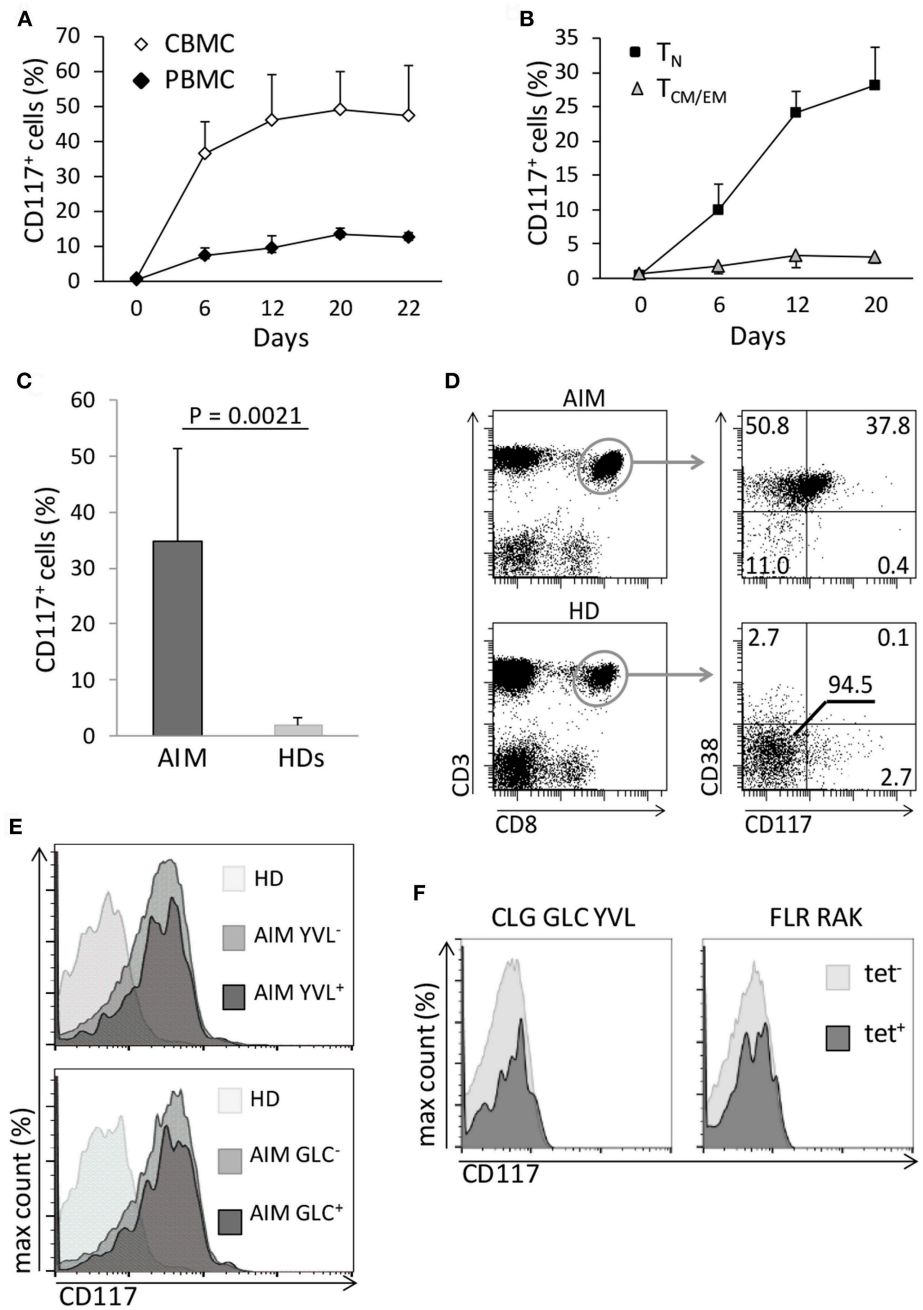
Only T_N_ have the potential to express CD117, and the phenomenon can occur *in vivo*. **(A)** CBMC and PBMC were activated and the percentage of CD8^+^ T cells expressing CD117 was recorded at the indicated time points. Data are from three independent samples. **(B)** Purified CCR7^+^/CD45RA^+^ CD8^+^ T_N_ and CD45RA^−^ CD8^+^ T_CM/EM_ from PB of adult donors were activated and CD117 expression recorded at the indicated time points. Data are from three independent samples. **(C)** Expression of CD117 was measured in PB CD8^+^ T cells from six patients with AIM and from six healthy donors (HDs). **(D)** Expression of CD117 and CD38 was measured in PB CD8^+^ T cells from three patients with AIM and from three HDs. Single representative experiment. **(E)** PBMC from three patients with AIM, different from those in **(D)** and all expressing HLA A2, and from three EBV-negative HD were stained with YVL (upper panel) and GLC (lower panel) tetramers. The expression of CD117 is shown in CD8^+^ T cells from an HD and in either tetramer-positive or tetramer-negative CD8^+^ T cells from an AIM patient. Single representative experiment. **(F)** PBMC from three EBV seropositive HDs expressing the appropriate HLA alleles were stained with tetramers specific for the HLA A2-restricted CLG, GLC, and YVL peptides (left panel) or for the HLA B8-restricted FLR and RAK peptides (right panel). The expression of CD117 is shown in CD8^+^ T cells from tetramer-positive and tetramer-negative CD8^+^ T cells. Single representative experiments.

As CD117 is typically expressed on early stem cell populations, and our initial studies had focused on mature T cells from UCB, we next went on to assess the expression of CD117 on CD8^+^ T cells from peripheral blood (PB) of adult donors. Blood samples were taken from three donors, aged 28–63 years, and stimulated *in vitro* as above. Of note, CD117 expression was also observed on CD8^+^ T cells from the adult population although this was seen at a much lower level in comparison to cells from UCB. In particular, CD117 was observed on 12% of CD8^+^ T cells at 3 weeks after stimulation compared to 45% of cells from UCB (Figure 3A). However, it is important to consider that adult peripheral blood mononuclear cells (PBMC) comprise large numbers of memory cells whilst T cells within CBMC are almost all CCR7^+^/CD45RA^+^ naïve (T_N_). Importantly, when the kinetic curve of CD117 expression was examined on different T memory subsets, minimal levels of CD117 were seen on CCR7^+^/CD45RA^−^ central memory (T_CM_) and CCR7^−^/CD45RA^−^ effector memory (T_EM_) CD8^+^ T cells from adult donors whereas expression on T_N_ populations reached 28% (Figure 3B). As seen in UCB, the expression of CD117 on activated CD4^+^ T cells from adult donors was negligible (data not shown). These studies show that activation-induced expression of CD117 is observed only on the naive subpopulation of CD8^+^ T cells.

In order to look for evidence of CD117 expression on CD8^+^ T cells *in vivo* we next examined populations of CD8^+^ T cells taken from donors after recent primary viral infection. Therefore, blood was taken from patients with acute infectious mononucleosis (AIM) due to recent Epstein-Barr virus (EBV) infection. CD117^+^ cells were found to represent 35% of the circulating CD8^+^ T cell pool in AIM patients (Figure 3C) and these cells also expressed CD38 (Figure 3D) a protein highly expressed by EBV-specific T cells during AIM (14). In addition, the proportion of CD117^+^ cells was examined on EBV-specific CD8^+^ T cells identified through staining with HLA-peptide tetramers. CD117 was again highly expressed on CD8^+^ T cells specific for the HLA A^*^0201-restricted, EBV-derived peptides YVLDHLIVV (YVL) and GLCTLVAML (GLC) (Figure 3E). Importantly, EBV-specific CD8^+^ T cells in EBV seropositive healthy donors did not express CD117 (Figure 3F).

### The Magnitude of Activation-Induced CD117 Expression Is Inversely Related to the Strength of T Cell Stimulation

**Figure 4:**
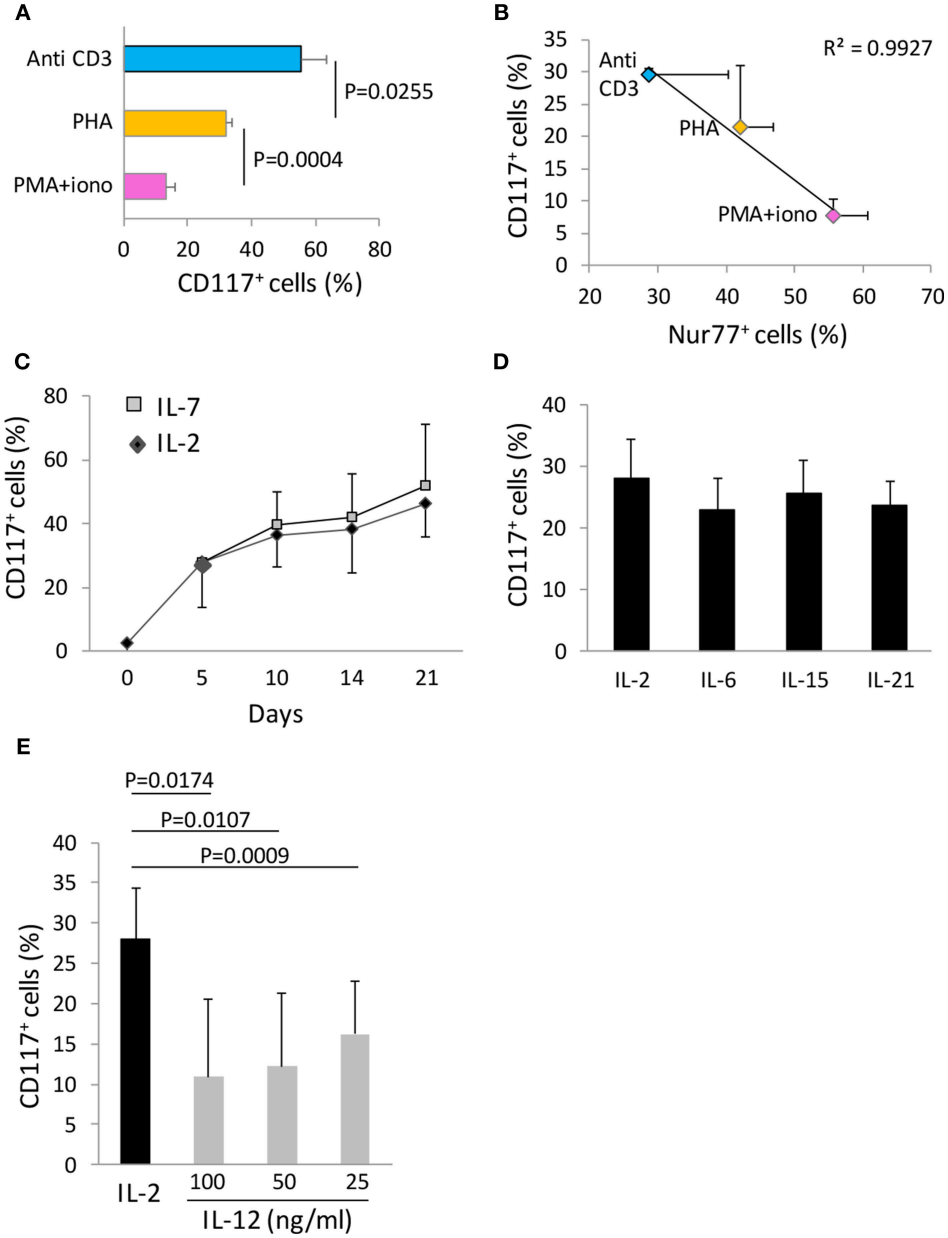
CD117 expression depends on cytokine milieu and the strength of the activating stimulus. **(A)** CBMC were activated with the indicated stimuli and the percentage of CD8^+^ T cells expressing CD117 was recorded at day 21. Data are from three independent samples. **(B)** CBMC were activated with the indicated stimuli and the percentage of CD8^+^ T cells expressing Nur77 at day 1 was plotted against the percentage of CD8^+^ T cells expressing CD117 at day 4. Data are from four independent samples. **(C)** Activated CBMC at day 5 were either maintained in IL-2 50 U/mL, or washed and maintained in IL-7 50 ng/mL. The percentage of CD8^+^ T cells expressing CD117 was recorded at the indicated time points. Data are from three independent samples.**(D)** Activated CBMC at day 5 were either maintained in IL-2 50 U/mL, or washed and maintained in IL-6, IL-15, or IL-21, each at the concentration of 50 ng/mL. The percentage of CD8^+^ T cells expressing CD117 at day 10 is shown. Data are from three independent samples. **(E)** Activated CBMC at day 5 were either maintained in IL-2 50 U/mL, or washed and maintained in IL-12 at the concentrations indicated. The percentage of CD8^+^ T cells expressing CD117 was recorded at day 10. Data are from three independent samples.

We next examined how the strength of the initial stimulus was related to the induction of CD117 expression on CD8^+^ T cells. Anti-CD3 antibodies, PHA, and PMA-ionomycin were selected to represent increasing strengths of T cell activation and were examined for their ability to induce CD117 expression on cord blood T cells. Interestingly, the level of activation-induced CD117 expression showed a progressive decrease with increasing intensity of T cell activation (Figure 4A). As such, CD117 expression was observed on 57% of cells after stimulation with anti-CD3 compared to only 12% of cells following stimulation with the potent mitogenic combination of PMA and ionomycin. To confirm that this inverse correlation was indeed reflective of the intensity of the initial stimulus we next assessed the expression of Nur77, a transcription factor whose expression has recently been shown to reflect the strength of activating signaling in human T cells (9). This confirmed that activation with soluble anti-CD3, PHA, or PMA-ionomycin led to induction of increasing levels of Nur77 expression but these were again inversely correlated with the subsequent level of CD117 expression (Figure 4B). These data show that the level of activation-induced CD117 expression is inversely related to the strength of the activating stimulus and as such higher levels are found on cells that have undergone less intense initial stimulation.

We were also able to investigate the importance of the cytokine microenvironment on the level of CD117 induction. IL-2 was added to all cultures at the time of initial activation and maintained or replaced by different cytokines from day 5 onwards. Replacement of IL-2 by IL-7 did not lead to any difference in the kinetics or magnitude of CD117 expression over a 21 day culture period (Figure 4C). Incubation with IL-6, IL-15, or IL-21 also did not influence the expression of CD117 compared to cells maintained in IL-2 (Figure 4D). However, it was noteworthy that replacement of IL-2 with IL-12 sharply reduced the level of CD117 expression at all doses examined between 25 and 100 ng/ml (Figure 4E).

### Activation-Induced CD117^+^/CD8^+^ Cells Express CD39 and Exhibit Markedly Increased Susceptibility to Apoptosis Compared to CD117—Cells

**Figure 5:**
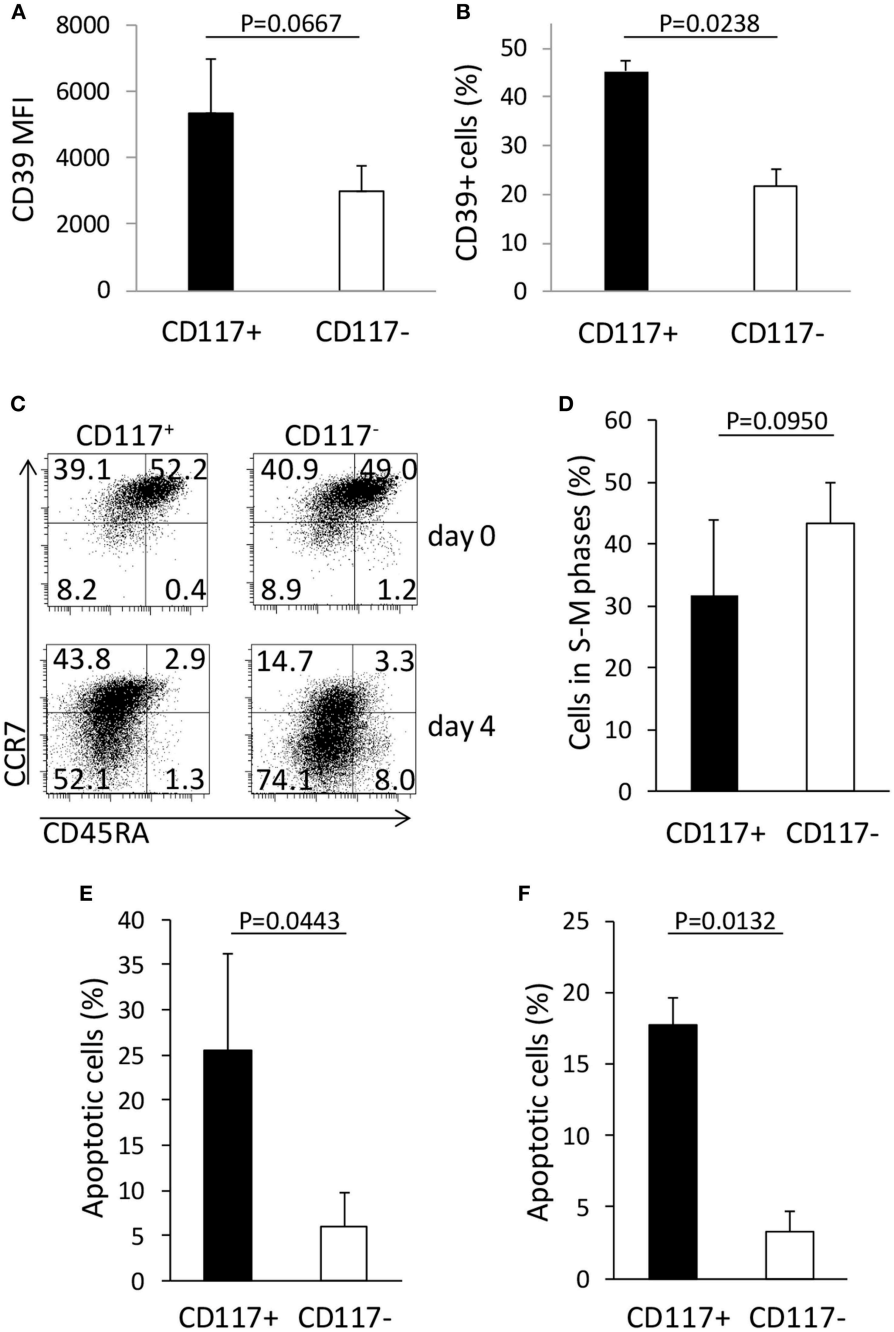
CD117 expression defines CD8^+^ T cells subsets with different phenotypic and functional characteristics. **(A,B)** CD39 expression in CD8^+^/CD117^+^ and in CD8^+^/CD117^−^ cells. The mean fluorescence intensity (MFI) of the positive population **(A)** and the percentage of the CD39^+^ cells **(B)** are shown for the two cell subsets. Data are from three independent samples. **(C)** The expression of CCR7 and CD45RA was measured in enriched CD117^+^ and CD117^−^ cells before and 4 days after re-stimulation. Single representative experiment out of three. **(D)** Enriched CD117^+^ and CD117^−^ cells were re-activated and the cells in the different phases of the cell cycle were measured at day 3 by evaluating the hyperdiploid DNA content via PI uptake. Data are from three independent samples. **(E,F)** Enriched CD117^+^ and CD117^−^ cells were re-activated and after 3 days dexamethasone 10^−6^ M was added. After 24 h the percentage of apoptosis was evaluated measuring the hypodiploid DNA content **(E)** or the binding of Annexin V **(F)**. Data are from three independent samples.

In order to interrogate further differences in the phenotype and function of CD117^+^ and CD117^−^ CD8^+^ cells we then used flow cytometry to examine the expression of a range of phenotypic markers comprising CD11a, CD11b, CD25, CD28, CD31, CD39, CD57, CD84, CD127, CD130, CD40L, CTLA-4, CCR2, CCR5, CCR9, KLRG-1, PD-1, TIM-3, and Integrin β7. Expression of these markers did not differ between the two groups with the exception of the ectonucleotidase CD39 (Figures S2B,C). As such the percentage of cells that expressed CD39 and the mean fluorescence intensity of CD39 expression were both increased on the CD117^+^ subset (Figures 5A,B).

In order to examine potential differences in functional response we next examined the profile of cytokine expression by the two cell subsets. This showed no difference in the level of IL-2, TNF-α, or IFN-γ expression by CD117^+^ and CD117^−^ cells after mitogenic stimulation (Figure S3A).

CD39 expression has been reported as a marker of T cell exhaustion of CD8^+^ T cells (15) and we therefore investigated the patterns of differentiation, proliferation, and response to apoptotic stimuli on CD117^−^ and CD117^+^ CD8^+^ T cell subsets. CD117^+^ and CD117^−^ cells derived from recently activated CD8^+^ T cells were isolated and then re-stimulated *in vitro*. Interestingly, CD117^−^ cells exhibited a more differentiated phenotype compared to the CD117^+^ subset (Figure 5C) and this correlated with a trend toward increased levels of mitosis in the CD117^−^ subset (Figure 5D). In order to assess comparative susceptibility to apoptosis, the two cell subsets were re-stimulated and after 3 days dexamethasone was added for 24 h prior to assessment of the proportion of cells that were undergoing apoptosis. The percentage of apoptotic cells was found to be markedly higher within the CD117^+^ subset compared to CD117^−^ cells. Indeed, when assessed by either hypodiploid DNA content or binding of Annexin V, around 20% of CD117^+^ cells had started to undergo apoptosis. This value was five times higher than levels seen within the CD117—subset (Figures 5E,F).

### Engagement of CD117^+^ CD8^+^ Cells With Membrane-Bound SCF Induces Increased Levels of Cell Death in Response to Apoptotic Stimuli

**Figure 6:**
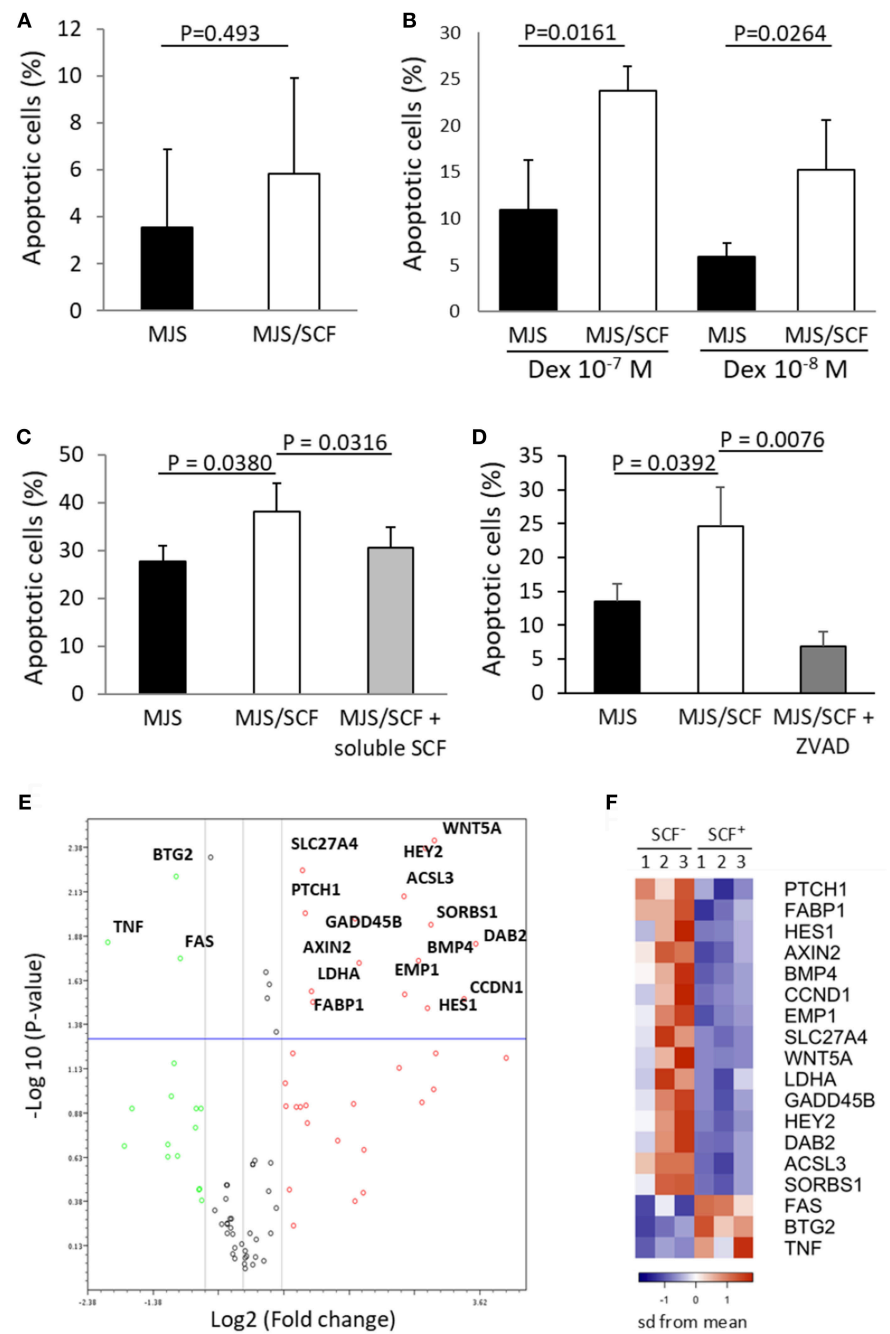
Triggering of CD117 delivers a pro-apoptotic signal through regulation of specific genes. **(A)** Activation-induced and enriched CD117^+^ cells were co-incubated with SCF^220^-transduced or mock-transduced MJS cells and apoptosis after 24 h was assessed by measuring hypodiploid DNA content. Data are from three independent samples. **(B)** Activation-induced and enriched CD117^+^ cells were co-incubated with SCF^220^-transduced or mock-transduced MJS cells in presence of dexamethasone for 24 h prior to assessment of apoptosis. Data are from three independent samples. **(C)** Activation-induced and enriched CD117^+^ cells were co-incubated with SCF^220^-transduced or mock-transduced MJS cells in presence of galectin-1 (10^−5^ M). Apoptosis was measured after 24 h. A subpopulation of CD117^+^ cells was pre-incubated with soluble SCF prior to co-incubation. Data are from three independent samples. **(D)** Activation-induced and enriched CD117^+^ cells were co-incubated with SCF^220^-transduced or mock-transduced MJS cells in presence of dexamethasone (10^−7^ M) with or without Z-VAD-FMK (10^−4^ M). Data are from three independent samples. **(E)** Enriched CD117^+^ cells were incubated overnight with MJS cells, either transduced with SCF^220^ or mock-transduced, in the presence of dexamethasone (10^−7^ M). MJS cells were then removed and expression of 84 genes representing canonical signaling pathways was determined. The plot shows genes that are upregulated (red) or downregulated (green) in CD117^+^ cells co-incubated with SCF^220^-transduced MJS cells. Data are from three independent samples. **(F)** Heat map showing the major differentially-expressed genes in CD117^+^ cells co-incubated with MJS cells transduced with SCF^220^ (SCF^+^) compared to mock-transduced cells (SCF^−^). The down-regulated genes in blue and up-regulated genes in orange.

In order to further evaluate the physiological significance of CD117 expression on CD8^+^ T cells we next examined the cellular response to engagement with soluble and cell-associated SCF ligand. Initially, recently activated CD8^+^ T cells were incubated with soluble SCF and this was seen not to influence the phenotypic differentiation of the CD117^+^ subset upon re-activation (data not shown). In addition, soluble SCF also did not modulate the proliferation of CD117^+^ cells (Figures S3B,C) or regulate the induction of apoptosis upon administration of dexamethasone (Figure S3D). Membrane-bound SCF provides a stronger form of ligand engagement compared to soluble SCF (1) and we therefore transfected the membrane-bound SCF^220^ isoform into cells from the melanoma cell line Mel JuSo (MJS) prior to assessment during co-culture with CD117^+^ cells. Indeed, enrichment of activation-induced CD117^+^ CD8^+^ T cells followed by reactivation and co-incubation with SCF^220^-transduced MJS cells alone did increase the rate of apoptosis in the former cells (Figure 6A), although no effect was observed on their proliferation (data not shown). However, when co-incubation was carried out in the presence of dexamethasone or galectin-1, both of which can induce apoptosis in CD8^+^ cells (16, 17), the proportion of apoptotic T cells was markedly higher when they were co-incubated with SCF^220^-transduced MJS cells (Figures 6B,C). This increase was blocked when CD117^+^ cells were pre-incubated with SCF which, as shown earlier, induces down-regulation of CD117 (Figure 6C). Similar inhibition was obtained when the pan caspase inhibitor Z-VAD-FMK was added to the co-cultures (Figure 6D).

### Engagement of CD117^+^ CD8^+^ T Cells With Membrane-Bound SCF Leads to Activation of a Range of Signaling Pathways Acting Through the Transcriptional Repressor HES1

In order to investigate the signal transduction pathways that are activated following CD117 engagement we next utilized signal transduction pathway finder PCR array to analyze responses within CD117^+^ cells co-incubated with membrane bound SCF in the presence of dexamethasone. Of the 84 key genes representative of 18 different signal transduction pathways, 15 were significantly up-regulated and three were significantly down-regulated (Figures 6E,F). Interestingly, activation of the Hedgehog, Wnt and Notch signaling pathways was observed and these all act through the transcriptional repressor HES1 whose expression was itself increased by 10-fold following c-Kit engagement (Tables S2, S3, and Figure 6D).

## Discussion

CD117 is expressed on many stem cell populations and engagement with its ligand SCF plays an important role in tissue homoeostasis and haemopoiesis. Here we show that CD117 expression is also induced on mature CD8^+^ T cells following initial activation. The identification of CD117 on differentiated somatic cells is unusual and to date expression within the haemopoietic lineage has been reported only on mast cells. Expression on recently activated CD8^+^ T cells was suggested by increased levels of CD117 mRNA and confirmed by staining with four different CD117-specific antibodies.

Our studies uncovered several interesting observations including the fact that CD117 was induced only on CD8^+^ T cells and not the CD4^+^ subset. The reasons for this are not yet clear but add to the understanding of the significant differences between these two major lineages of the adaptive immune response. In addition, CD117 was expressed only at the time of the initial activation of CD8^+^ T cell and not when memory T cells underwent re-stimulation. This suggests that CD117 expression has evolved to play an important role in shaping the profile of the initial CD8^+^ repertoire in response to antigen engagement. CD117 expression was intrinsic to CD8^+^ T cells and did not require inductive signaling from other cells although IL-12 acted to suppress CD117 induction, a finding consistent with the anti-apoptotic role for IL-12 in promoting survival and expansion of naive CD8^+^ T cells following initial engagement (18, 19).

An important aspect of our study was that we were able to identify CD117 expression on recently activated CD8^+^ T cells *in vivo* in patients suffering from AIM, a condition seen following primary infection with EBV and associated with intense activation of EBV-specific CD8^+^ T cells (14). The activation marker CD38 is expressed on the majority of EBV-specific cells in AIM and CD117^+^ expression was seen on 41% of CD38^+^/CD8^+^ cells. In order to confirm that this pattern was associated with the EBV-specific CTL we also incorporated HLA-peptide tetramers and documented CD117 expression on virus-specific populations. EBV establishes a state of chronic infection associated with a substantial virus-specific immune response and it was noteworthy that EBV-specific CD8^+^ T cells in this setting did not display CD117 expression, further supporting our *in vitro* data that CD117 expression is restricted to activation of naïve cells.

These observations suggest that CD117 expression by CD8^+^ T cells might also represent an important biomarker in a range of clinical settings such as cancer immunotherapy or vaccine response. Examples might include assessment of the induction of immunogenic cell death or optimization of radiotherapy scheduling to achieve an abscopal effect.

CD117 expression was induced on only a subset of recently activated CD8^+^ T cells and led us to investigate the functional differences between CD117^−^ and CD117^+^ subpopulations. CD117 engagement on stem and precursor cells is typically associated with expansion and differentiation (1, 2) and we therefore anticipated that it might also promote these qualities on CD8^+^ T cells. Unexpectedly, CD117^+^ CD8^+^ T cells underwent less proliferation and differentiation compared to the CD117^−^ subset and the most striking effect was that CD117 engagement strongly promoted cell death in the presence of pro-apoptotic stimuli.

Interestingly, CD117 expression on tumor cells is lost during progression of melanoma and enforced expression followed by SCF engagement triggers apoptosis both *in vitro* and *in vivo* (20). As such CD117-SCF engagement appears to have differential effects on cellular function in stem cell and somatic tissues. Of note, the only phenotypic difference between CD117^+^ and CD117^−^ T cells was in relation to expression of the ectonucleotidase CD39, which has also been associated with increased susceptibility to apoptosis in T cells (21, 22) and with T cell senescence (23).

SCF is expressed by stromal cells in bone marrow, thymus and lymph nodes (24, 25), intestinal epithelial cells (7), cells in the dermis and testis (26, 27) as well as neurons, mast cells, and activated dendritic cells (28–31). As such, SCF expression may potentially act to regulate CD8^+^ T cell repertoire formation in a wide variety of anatomical sites.

We observed that, in the presence of pro-apoptotic stimuli, CD117 engagement on CD8^+^ T cells upregulated many proteins, such as PTCH1, WNT5A, AXIN2, BMP4, and HEY2, and suggests involvement of the Hedgehog, Wnt and Notch signaling pathways. Importantly, these pathways converge on the transcriptional repressor HES1 (32), which was upregulated 25-fold following CD177 engagement, and acts as an important mediator of Notch-mediated apoptosis (33). HES1 is a critical factor in the specification and commitment of T cells (34) and these data suggest that it also regulates the early survival of mature CD8^+^ T cells. In further accordance with the pro-apoptotic effect resulting from CD117 triggering, non-canonical Wnt signaling via Wnt5a increases apoptosis in T cells (35) and Gadd45b and Dab2 also mediate pro-apoptotic signals (36, 37). In contrast, downregulation of Fas is likely to be less important as recently activated T cells are resistant toward Fas-mediated apoptosis (38). Caspases likely play a role in mediating the amplification of pro-apoptotic stimuli by SCF.

A striking finding was that the level of CD117 expression was inversely related to the strength of initial cell stimulation. As such, the intensity of CD117 expression acts to stratify recently activated naïve cells according to their level of initial engagement and sensitizes this pool with differential sensitivity to apoptosis. This effect would be expected to act to “cleanse” the initial T cell proliferative pool of cells that had undergo weak interactions with cognate antigen and thus increase the median affinity of the effector population. Indeed, although T cell receptor genes do not undergo somatic mutation the functional avidity of primary T cell responses can increase >50-fold during the early stages of viral infection (39, 40) and CD117-mediated purging of the primary repertoire may contribute to this effect. It is interesting to contrast the potential role of CD117 expression with that of Fas (CD95) which can also induce apoptosis. Contrary to CD117, the expression of Fas does not appear to be related to the strength of activation and Fas engagement acts to deliver a primary apoptotic signal in contrast to CD117-mediated enhancement of concomitant pro-apoptotic stimuli.

However, in addition to this physiological role, CD117 expression on CD8^+^ T cells may also be an important factor in tumor immune evasion. The accumulation of somatic mutations during tumor progression drives the presentation of peptide neoantigens and subsequent T cell-mediated recognition is thought to play an important role in limiting disease progression. As neoantigens are derived from endogenous peptides they typically make low affinity interactions with the host adaptive immune response (41) and our data would suggest that this would be reflected in significant CD117 expression on tumor-specific T cells. Importantly, we observed that galetin-1 markedly enhanced the apoptosis of recently activated CD117^+^/CD8^+^ T cells when these underwent engagement with cell-associated SCF. Galectin-1 is expressed in a wide range of tumors (42–47) and its expression correlates with disease progression (42, 45). Many tumors also express SCF (28, 48–51) and CD117, generating an autocrine loop that supports tumor growth (48, 49) and providing the conditions required for CD117-mediated amplification of T cell apoptosis. Indeed, targeting of galectin-1 overcomes tumor-associated immune suppression in a mouse model of breast cancer (52). The activation of pro-apoptotic mechanisms on T cells plays an important role in immune evasion in cancer and engagement of CD117 may represent an additional such pathway. Indeed, inhibition of CD117 by a specific antibody enhances the effect of immune checkpoint inhibitors on the growth of syngeneic mouse tumors and of canine spontaneous mast cell tumors (53, 54). The authors hypothesize that the effect of CD117 blockade likely reflects a specific role of CD117 in modulating the immune system; herein we provide evidence for this role.

Our findings reveal that CD117, a receptor whose expression had typically been thought to be restricted to stem cells, is also expressed on mature CD8^+^ T cells following initial activation. In direct contrast to its role in supporting proliferation and differentiation of early progenitors, CD117 on CD8^+^ T cells acts to suppress differentiation and increase sensitivity to apoptosis. Expression is therefore likely to play an important role in shaping CD8^+^ T cell immunodominance and CD117-blockade, potentially in combination with checkpoint inhibition, may represent a novel form of immunotherapy to increase the breadth and affinity of tumor-specific CD8^+^ immune responses.

## Data Availability

Microarray data have been deposited in Gene Expression Omnibus, accession number GSE114812. The gene pathway dataset was published on Mendeley doi: 10.17632/9nwk7xk5pd.1.

## Ethics Statement

The study was approved by the National Research Ethics Committee, UK REC no. 11/WM/0315, and by the Non-Clinical Issue committee of the NHS Blood and Transplant. Human UCB from anonymized collections, unsuitable for hematopoietic stem cell transplant, was provided by the NHS Cord Blood Bank, UK, as Non-Clinical Issue. PB was collected from consenting patients with AIM and from adult healthy blood donors from the NHS Blood and Transplant Donor Center, Birmingham, UK. All donors gave written informed consent to authorize the use of their blood for medical, pharmaceutical, and research purposes.

## Author Contributions

GF, FC, and PM developed the concept. GF, JZ, and KV designed the experiments. GF and KV performed experiments. JZ generated the vector for transduction. GF, KV, PR, and WC assembled data, generated figures, and ran statistical analysis. FC and PM gave financial support. All the authors contributed to writing the manuscript.

### Conflict of Interest Statement

The authors declare that the research was conducted in the absence of any commercial or financial relationships that could be construed as a potential conflict of interest.

## Abbreviations

AIM: acute infectious mononucleosis
PMA, phorbol 12-myristate 13-acetate: SCF: stem cell factor.
