# Supplementary material for: CD117 (c-Kit) Is Expressed During CD8+ T Cell Priming and Stratifies Sensitivity to Apoptosis According to Strength of TCR Engagement
Source: Front Immunol. 2019 Mar 15;10:468. doi: 10.3389/fimmu.2019.00468 (PMC6428734; doi:10.3389/fimmu.2019.00468)
Supplement: Supplementary file 1 [file Data_Sheet_1.docx]

**SUPPLEMENTARY MATERIAL**

| **Supplementary table 1. List of the other antibodies used for flow cytometry** | | | | | |
| --- | --- | --- | --- | --- | --- |
| **Antibody used** | **Clone** | **Company** | **Antibody used** | **Clone** | **Company** |
| CCR2/PE | K036C2 | BioLegend | CD38/PE-Cy7 | HB7 | BD |
| CCR5/PE-Cy7 | 2D7/CCR5 | BD | CD39PE/Cy7 | eBioA1 | eBioscience |
| CCR7/Fitc | G043H7 | BD | CD4/Pacific Blue | RPA-T4 | BD |
| CCR9/PE-Cy7 | L053E8 | BioLegend | CD45RA/PE-Cy7 | HI100 | BD |
| CD11a/PE | HI111 | BD | CD57/APC | HCD57 | BioLegend |
| CD11b/PE | ICRF44 | BD | CD8/APC-Cy7 | SK1 | BD |
| CD127/PE | A019D5 | BioLegend | CD84/PE | CD84.1.21 | BioLegend |
| CD130/Fitc | B-R3 | Bio-Rad | CD95/APC | DX2 | BD |
| CD14/PerCP | 134620 | R&D | CD40L/Fitc | 40804 | R&D |
| CD16/PerCP | 245536 | R&D | CTLA-4/PE | BNI3 | BD |
| CD19/PerCP | 4G7-2E3 | R&D | Integrin b7/PE | 473207 | R&D |
| CD25/APC | M-A251 | BD | KLRG-1/PE-Cy7 | 2FI/KLRG1 | BioLegend |
| CD28/APC | CD28.2 | BD | NGFR/APC | ME20.4 | BioLegend |
| CD3/V500 | UCHT1 | BD | PD-1/APC | PD1.3.1.3 | Miltenyi |
| CD31/APC-eFluor 780 | WM-59 | eBioscience | Tim-3/PE-Cy7 | F38-2E2 | BioLegend |

| **Supplementary Table 2. Pathways enriched in the upregulated genes** | | |
| --- | --- | --- |
| **Hallmark** | **pvalue** | **FDR** |
| NOTCH Signaling | 1.17E-06 | 5.85E-05 |
| WNT β catenin signaling | 2.70E-06 | 6.74E-05 |
| Apoptosis | 1.50E-04 | 2.04E-03 |
| Hedgehog signaling | 2.44E-04 | 2.04E-03 |
| Epithelial mesenchymal transition | 2.77E-04 | 2.04E-03 |
| TNFα signalling via NFKB | 2.86E-04 | 2.04E-03 |
| IL2 STAT5 signaling | 2.86E-04 | 2.04E-03 |
| Androgen response | 2.02E-03 | 1.26E-02 |
| MTORC1 signaling | 7.52E-03 | 3.49E-02 |
| Estrogen early response | 7.60E-03 | 3.49E-02 |
| Myogenesis | 7.67E-03 | 3.49E-02 |

| **Supplementary Table 3. Pathways enriched in the downregulated genes** | | |
| --- | --- | --- |
| **Hallmark Pathway** | ***p-value*** | **FDR** |
| Apoptosis | 1.75E-06 | 8.73E-05 |
| IL6-JAK-STAT3 signaling | 0.000102006 | 0.002550162 |
| P53 pathway | 0.000528028 | 0.005387073 |
| TNFa signalling via NFKB | 0.000533354 | 0.005387073 |
| Allograft rejection | 0.000538707 | 0.005387073 |
| Peroxisome | 0.01954864 | 0.117216708 |
| Bile acid metabolism | 0.021243049 | 0.117216708 |
| UV response | 0.029308267 | 0.117216708 |
| Fatty acid metabolism | 0.029682176 | 0.117216708 |
| KRAS signaling | 0.036580033 | 0.117216708 |
| Heme metabolism | 0.036765949 | 0.117216708 |
| Epithelial mesenchymal transition | 0.037137702 | 0.117216708 |
| Inflammatory response | 0.037137702 | 0.117216708 |
| IFNg response | 0.037323538 | 0.117216708 |
| MTORC1 signaling | 0.037323538 | 0.117216708 |
| Xenobiotic metabolism | 0.037509347 | 0.117216708 |

**Figure S1.** **(A)** MJS cell were transduced with constructs coding for NGFR only or NGFR and SCF^220^. The efficiency of transduction was measured by flow cytometry. One representative experiment is shown. **(B)** Cell proliferation and apoptosis were measured evaluating the cell DNA content via PI uptake. Two explicative histograms are shown.

**Figure S2.** **(A)** Freshly isolated CBMC were stained with the anti CD117 MoAbs indicated. Histograms show the results of gating on CD3^+^/CD8^+^ cells. Single representative experiment out of three. **(B)** CBMC were activated and at day 6 stained for the indicated markers. CD3^+^/CD8^+^ cells were gated, and the staining was evaluated on either CD117^+^ or CD117^-^ cells. Single representative experiment out of three. **(C)** The histograms from CD39 staining of CD117^+^ and CD117^-^ CD8^+^ T cells are shown. Single representative experiment out of three.

**Figure S3.** **(A)** Enriched CD117^+^ and CD117^-^ CD8^+^ T cells were re-activated with PMA-ionomycin, then stained for the intracellular content in IL-2, TNF and IFN **(B) **Enriched CD117^+^ cells were re-stimulated in the presence of escalating doses of soluble SCF. Cell proliferation was measured by CFSE staining. Single representative experiment out of three. **(C)** Enriched CD117^+^ cells were re-stimulated in the presence of escalating doses of soluble SCF. Cell proliferation was evaluated by measuring hyperdiploid DNA content. **(D)** Enriched CD117^+^ cells were re-activated and after three days dexamethasone 1 μM was added, in the presence of escalating doses of soluble SCF. After 24 hours the percentage of apoptosis was evaluated measuring the hypodiploid DNA content. Data are from three experiments.
